# Beliefs and Determinants of Use of Traditional Complementary/Alternative Medicine in Pediatric Patients Who Undergo Treatment for Cancer in South America

**DOI:** 10.1200/JGO.2016.006809

**Published:** 2017-02-08

**Authors:** Valeria Rocha, Elena J. Ladas, Meiko Lin, Walter Cacciavillano, Elizabeth Ginn, Kara M. Kelly, Guillermo Chantada, Luis Castillo

**Affiliations:** **Valeria Rocha** and **Luis Castillo**, Centro Hospitalario Pereira Rossell, Montevideo, Uruguay; **Elena J. Ladas**, **Meiko Lin**, and **Elizabeth Ginn**, Columbia University Medical Center, New York; **Kara M. Kelly**, Roswell Park Cancer Center, Buffalo, NY; and **Walter Cacciavillano** and **Guillermo Chantada**, Hospital J.P. Garrahan, Buenos Aires, Argentina.

## Abstract

**Purpose:**

The use of traditional complementary/alternative medicine (TCAM) among children with cancer has been well documented. South America has a rich history of traditional healers and medicinal resources; however, little is known about the use of TCAM among children with cancer. We sought to investigate patterns, beliefs, and determinants of TCAM use among South American children with cancer.

**Methods:**

A cross-sectional survey was administered to 199 children treated for cancer at institutions located in Buenos Aires, Argentina, and Montevideo, Uruguay. Participants were queried about the type of TCAM and strength of beliefs associated with its use. Logistic regression analysis was used to estimate the odds ratios with 95% CIs.

**Results:**

We found that the use of TCAM was common in both Argentina (47%) and Uruguay (76%). Variations in the forms of TCAM used were observed between the countries; however, both countries used TCAM primarily for supportive care. Mother’s education, wealth index, and TCAM belief system were significant predictors of TCAM.

**Conclusion:**

To our knowledge, this study is the first to report on the use of TCAM in pediatric oncology in South America. The study identifies several predictors of TCAM use, which may serve as target variables for educational and research initiatives. The finding that most families use TCAM for supportive care suggests that future efforts could evaluate the role of TCAM to enhance existing supportive care regimens, particularly in settings where access to conventional medications are limited.

## INTRODUCTION

Complementary and alternative medicine is defined by the National Center for Complementary and Integrative Health as diverse medical health care systems, practices, and products that are not presently considered to be part of conventional medicine.^1^ Several studies have documented a high use of traditional complementary/alternative medicine (TCAM) among children with cancer, with many children and their families choosing to supplement with herbal or dietary supplements alongside cancer treatment.^2^ A recent review that has documented the prevalence of TCAM in pediatric oncology found that TCAM use is persistently and significantly higher among children who receive treatment in low- and middle-income countries compared with high-income countries.^3^

The Latin American Pediatric Oncology Group has fostered improvements in survival among children who undergo cancer treatment in South America.^4^ Argentina and Uruguay have strong cultural traditions and have become high-income countries, which enables them to project a different profile from that of most Latin American countries. Argentina^5^ and Uruguay^6,7^ have a strong Spanish and Italian cultural tradition, with a proportion culturally influenced by the indigenous populations, the Iberian Peninsula, Europe, and Africa.

The use of TCAM among children with cancer in South America is poorly documented in Argentina^8-11^ and Uruguay.^12,13^ In both countries, there is not an official policy governing the use of TCAM; however, both countries have regulated some forms of TCAM. Nevertheless, a few public and private hospitals offer some kind of TCAM, but documentation and inquiry about its use during clinical visits are not routine. More information is needed to understand the unique factors inherent to Argentina and Uruguay and their influence on TCAM use so that effective educational and clinical interventions can be designed and tested. As part of an international partnership with the Asociación de Hemato-Oncología Pediátrica de Centro América, we define the prevalence of use, determinants, and associated beliefs of TCAM in South American children and adolescents with cancer.

## METHODS

We conducted a survey in a cross-sectional sample of 199 children and adolescents with a new diagnosis of cancer and who were undergoing treatment, had completed treatment, had relapsed, or were receiving palliative care and being followed in the Centro Hospitalario Pereira Rossell in Montevideo, Uruguay (February through July 2013), and Hospital J.P. Garrahan in Buenos Aires, Argentina (February 2011 through November 2011). Approval was obtained from the institutional review boards at Columbia University Medical Center and the participating centers.

Children and adolescents 10 to 19 years of age were selected according to a computer-generated randomization code and were approached about participation during a routine visit to the inpatient or outpatient center. Informed consent was obtained from the parent or legal guardian for children younger than 18 years of age. Assent was obtained from children 7 years of age and older. The survey was then administered face to face by a trained interviewer from the local community who was not involved in the care of the child. To establish test-retest reliability, a random sample of participants (20%) was administered a retest survey within 2 weeks of the initial survey.

The survey tool was adapted and validated from a previously administered survey from the Columbia University Medical Center, the lead institution.^14^ Briefly, the survey consists of four sections that collect information on health behaviors, health beliefs, use and type of TCAM, and demographics. TCAM therapies were identified by modalities described by the National Center for Complementary and Integrative Health, published literature on TCAM in Argentina and Uruguay, and institutional experience with TCAM. For each TCAM therapy, additional details were collected that included TCAM indication, cost, satisfaction, and perceived effectiveness. Dietary practices were categorized as conventional (standard of care) or TCAM. Prayer was considered an independent TCAM therapy when practiced specifically for health purposes. Demographic information was abstracted from the patient’s medical chart (age, sex, clinical characteristics). Ethnicity, religion, and education were obtained from self-report in the survey.

The Demographic and Health Survey was used as an indicator of wealth status.^15^ The wealth index was calculated from data collected on a household’s possession of selected assets (eg, televisions, refrigerators, telephones, motorcycles, radios, cars, types of water access, Internet access). The wealth index was then generated as a composite variable through principal components analysis. The continuous scale of wealth index was categorized into five equal quintiles from poorest (1) to wealthiest (5).

The health beliefs portion of the survey collected information on respondents’ strength of beliefs in TCAM theories about causes of symptoms, adverse effects, and the diagnosis of cancer by using a previously described methodology.^16^ Beliefs in TCAM therapies included five items (eg, TCAM has fewer adverse effects than Western therapies, TCAM will be good for cancer treatment, TCAM is less effective than Western-style medicine) measured with a 5-point Likert scale and summarized as a composite score. This information was collected only in Uruguay because of their addition after completion of the survey in Argentina.

Demographic characteristics were summarized as mean, standard deviation, median, and range for continuous variables and as counts and percentages for nominal variables. The demographic information was compared between TCAM users and non-TCAM users by using either a two-sample *t* test for continuous variables or a χ^2^ test for categorical variables.

For the TCAM therapies construct, test-retest reliability was measured in 20% of respondents, who were retested by the same interviewer within 7 days of administration of the first survey. Agreeability among responses was measured with the intraclass correlation coefficient (ICC). An ICC value < 0.40 was considered poor agreement, a value between 0.40 and 0.75 was considered moderate agreement, and a value > 0.75 was considered excellent agreement.

To determine the difference between Argentina and Uruguay in selected TCAM prevalence variables (type of TCAM therapies used, cost of TCAM therapies, and perceived effectiveness of TCAM therapies), a χ^2^ test or Fisher’s exact test was conducted. A Mann-Whitney *U* test was performed to compare the number of TCAM therapies used by country.

To explore the relationships between TCAM use and demographic characteristics and beliefs in TCAM theories (both as composite scores and as quintiles), logistic regression analysis was used to estimate the odds ratios (ORs) with 95% CIs. Finally, to assess the influence of beliefs in TCAM theories (both as composite scores and as quintiles) on number of TCAM therapies used, Poisson distribution with robust error estimation was used. Statistical analysis was conducted with Stata 14 (StataCorp, College Station, TX) and SPSS version 20 (IBM Corporation, Chicago, IL) software. *P* ≤ .05 was considered statistically significant.

## RESULTS

### Demographics

One hundred and ninety-nine participants from Argentina (n = 99) and Uruguay (n = 100) participated in the survey (Table 1). Both countries were represented by slightly more males (Argentina, 57%; Uruguay, 58%); median age was similar between countries (Argentina, 7.3 [range, 0.7 to 18.6] years; Uruguay, 9 [range, 0.8-24.4] years). The majority of diagnoses were solid tumors and leukemia/lymphoma, which reflected the referral patterns of the institutions rather than the variation in incident cases between the two countries. In both countries, the majority of participants were undergoing treatment at the time of survey completion. Among Argentinean participants, significant differences between users and nonusers of TCAM were observed for family’s wealth index (*P* = .013) and father (*P* = .016) and mother’s level of education (*P* = .045). In Uruguay, significant differences were observed for age (*P* = .040) and mother’s level of education (*P* = .051). We did not find significant differences in the use of TCAM on the basis of the participants’ diagnoses in either country.

**Table 1:**
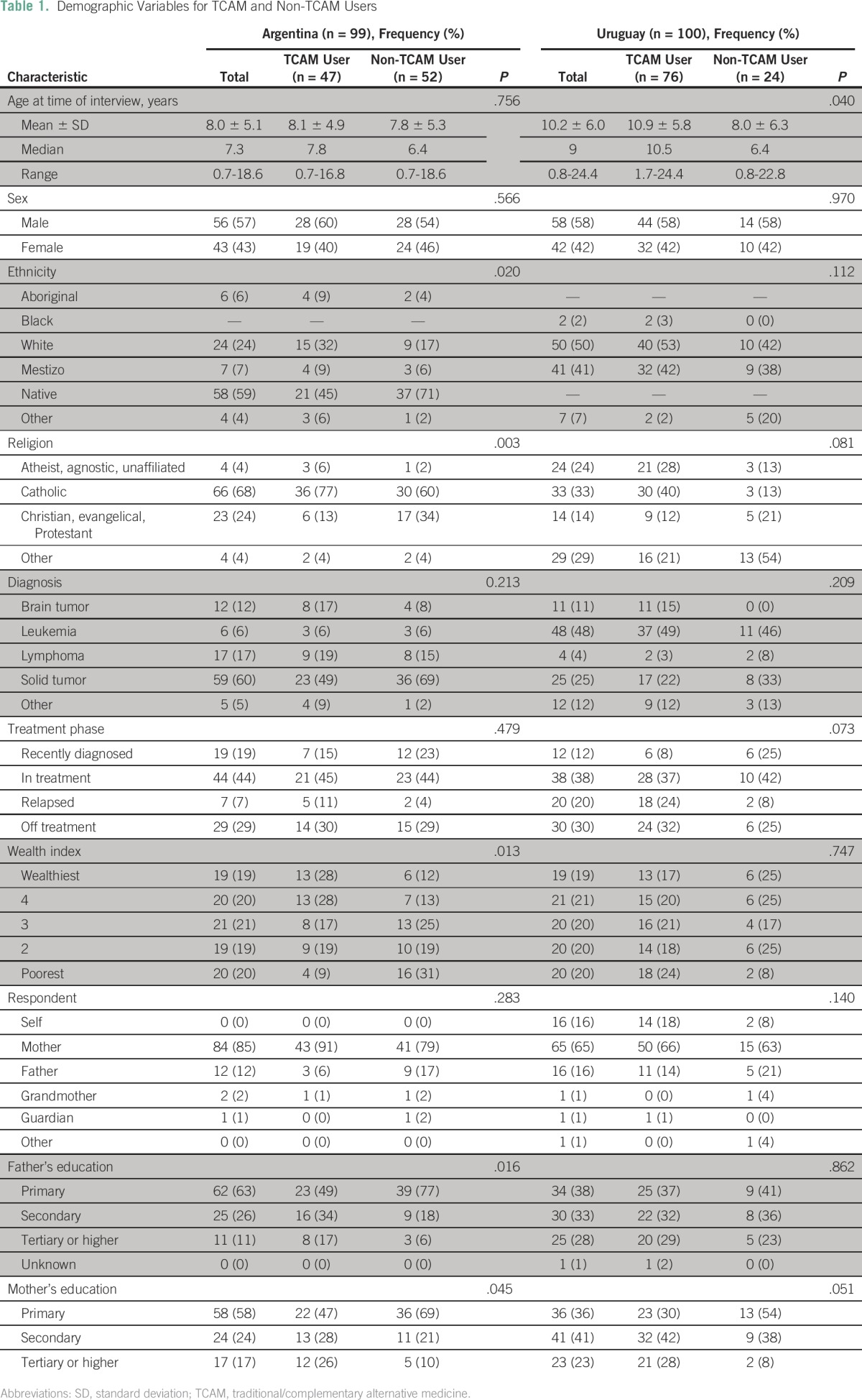
Demographic Variables for TCAM and Non-TCAM Users

### Prevalence of TCAM

For the TCAM therapies construct, high test-retest reliability was observed in Argentina (ICC, 0.977) and Uruguay (ICC, 0.866). Overall, we found a higher prevalence of the use of TCAM among Uruguayan (76%) than among Argentinean (47%) participants. Despite their proximity, we also found significant differences in the forms of TCAM used between the two countries (Table 2). Among Argentinean participants, the most frequently used therapies were diet change (21%), energy healing (14%), nutritional oral supplements (9%), and touch healing (8%). Among the Uruguayan participants, diet change (57%); energy healing (22%); herbal, botanical, teas, or other plant-based medications (17%); and touch or manual healing (11%) were the most commonly reported therapies. Appendix Table A1 lists the specific types of diet, dietary and herbal supplements, and ointments reported by the study participants.

**Table 2:**
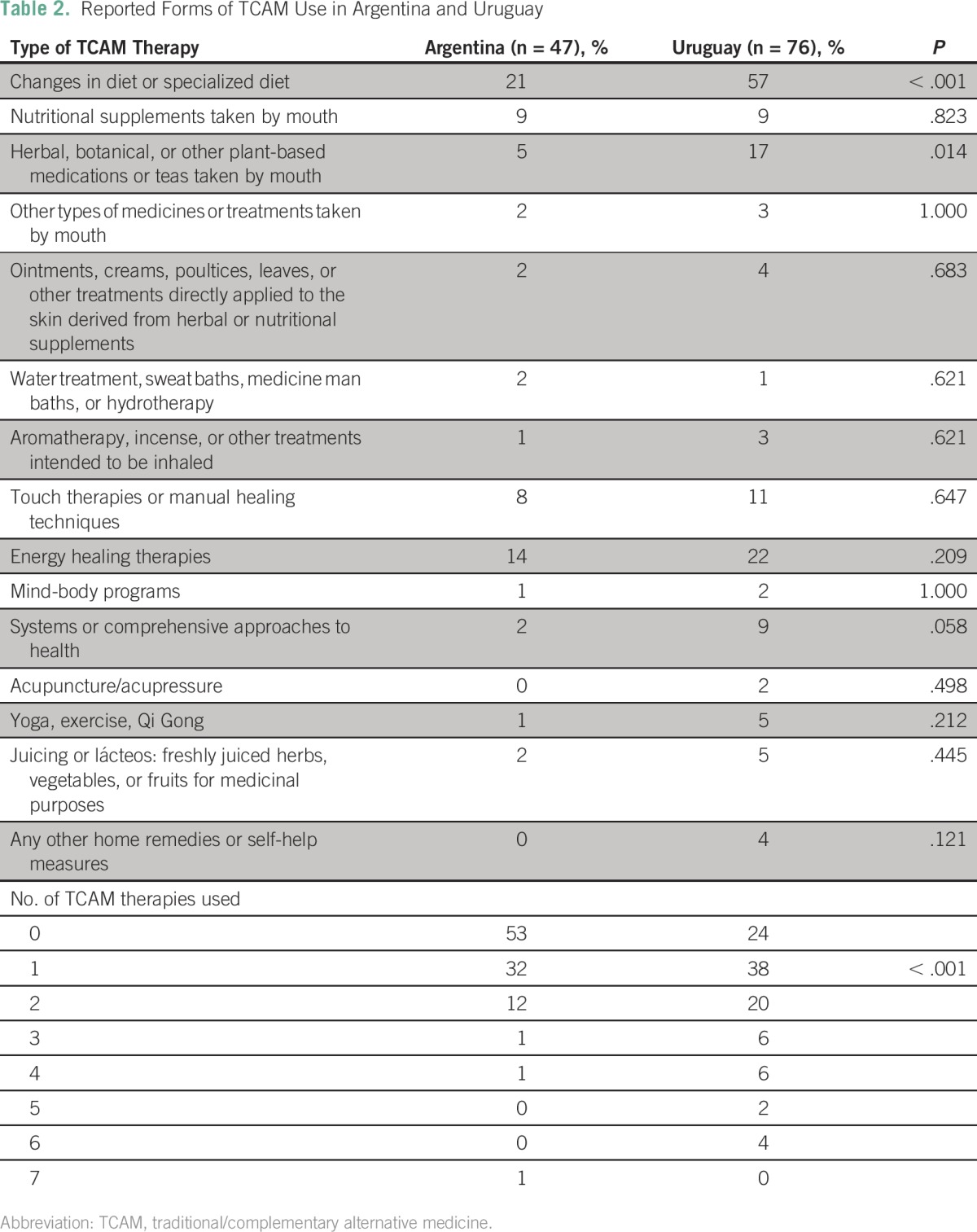
Reported Forms of TCAM Use in Argentina and Uruguay

In Uruguay, a significantly higher proportion of participants changed their diet (57%; *P* < .001) and consumed herbal supplements (17%; *P* = .014) than the Argentinean participants. We also found that a significantly higher proportion of participants used several forms of TCAM therapies in Uruguay than in Argentina (*P* < .001; Table 2). The cost of TCAM therapies may have contributed to the differences between countries because 57% of Argentinean participants reported free access to TCAM compared with 72% of Uruguayan participants.

A high proportion of therapies were considered effective or very effective in both Argentina (66%) and Uruguay (75%). The majority of participants reported their use of TCAM to their pediatric oncologist (Argentina, 61%; Uruguay, 55%).

### Reasons for TCAM Use

Despite variations in the prevalence and forms of TCAM, participants from both countries stated overall strengthening and well-being as a frequent reason for TCAM use (Argentina, 37%; Uruguay, 25%). Argentinean participants reported faith (23%) and to help cure their child (14%) as reasons for use of TCAM. In contrast, Uruguayan participants reported use of TCAM primarily for a variety of supportive care indications (63%), and a small proportion sought TCAM for curative purposes (5%).

### Determinants of TCAM Use

For both countries, we found that the mother’s level of education was a significant predictor of the use of TCAM. Children with college-educated mothers were more likely to use TCAM than those whose mothers had primary education or less (Argentina: OR, 3.93; 95% CI, 1.22 to 12.66; *P* = .022; Uruguay: OR, 5.94; 95% CI, 1.20 to 29.45; *P* = .029). The father’s educational level was a significant predictor in Argentina (OR, 4.52; 95% CI, 1.09 to 18.77; *P* = .038) only.

In Uruguay, demographic variables were associated with the number of TCAM therapies used. With each level of higher educational attainment of the mother was an increase in the number of TCAM therapies used (*P <* .001). Affiliation with Catholicism was also associated with more TCAM therapies (*P* < .001).

**Fig 1:**
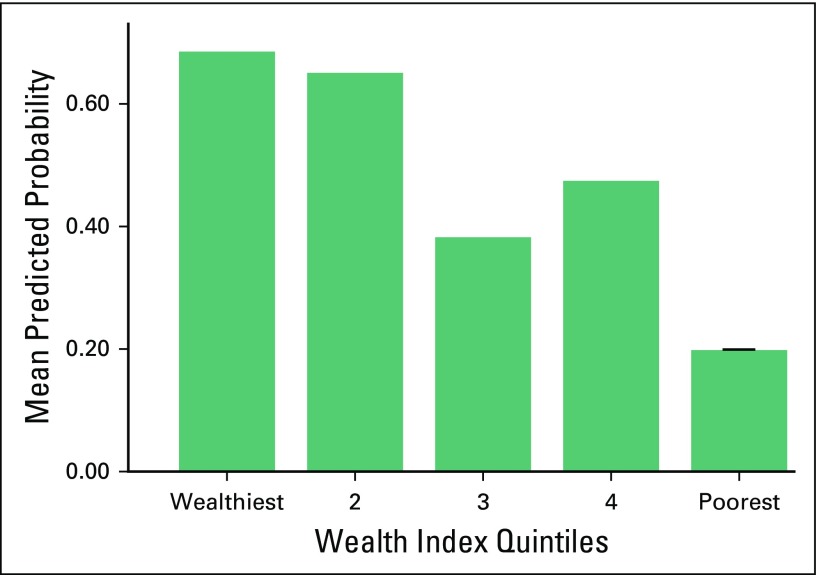
Logistic regression model of traditional/complementary alternative medicine by wealth quintiles (Argentina).

**Fig 2:**
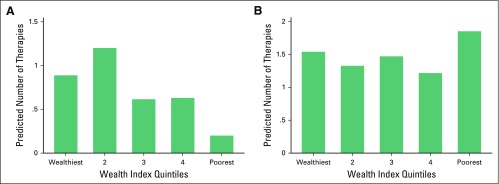
Number of traditional/complementary alternative medicine therapies used by wealth quintile in (A) Argentina and (B) Uruguay.

We found that the wealth quintile was a statistically significant predictor of use of TCAM among Argentinean participants (*P* = .010; Fig 1) but not Uruguayan participants (*P* = .415). Participants from the wealthiest households had an 8.67 times higher odds of using TCAM than those from lower wealth quintiles (OR, 8.67; 95% CI, 2.01 to 37.38; *P* = .004). Participants in the fourth wealth quintile, on average, had a seven times higher odds of using TCAM than those living in the lowest wealth quintile (OR, 7.43; 95% CI, 1.78 to 31.04; *P* = .006). In both countries, the wealth quintile was a significant predictor of the number of TCAM therapies used (*P* < .001 for both; Figs 2A and 2B).

Among Uruguayan participants, the family’s previous use of TCAM was a statistically significant predictor of TCAM use during therapy (*P* = .008). Families who had used TCAM in the past had a 5.90 times higher odds than those who had not (OR, 5.90; 95% CI, 1.26 to 27.57; *P* = .024). This observation was also found among adolescents and young adults when they served as the respondent (*P* = .040). Adolescents who used TCAM in the past had a 3.42 times higher odds than those who did not (OR, 3.42; 95% CI, 0.93 to 12.60; *P* = .063). For Argentina, the association between the family’s previous use of TCAM and the patient’s current use of TCAM approached significance (*P* = .061).

### Beliefs in TCAM (Uruguay Only)

**Fig 3:**
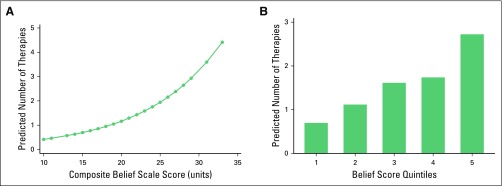
(A) Logistic regression model of traditional/complementary alternative medicine (TCAM) therapy use by parental TCAM belief scale (Uruguay). (B) Number of TCAM therapies used by parental TCAM belief scale (Uruguay).

The strength of beliefs in TCAM theories was significantly related to use of TCAM among the Uruguayan participants. Overall, the strength of parental beliefs in TCAM therapies was a significant predictor of the use of TCAM (*P* = .045). For every unit of increase on the parental belief scale, the odds of TCAM therapy use increased by a factor of 1.08 (OR, 1.08; 95% CI, 1.00 to 1.17; *P* = .052; Fig 3A). A similar observation was found for the patient belief scores (*P* = .016). For every unit of increase on the patient belief scale, the odds of TCAM therapy use increased by 1.13-fold (OR, 1.13; 95% CI, 1.02 to 1.26; *P* = .020). Parental belief scale quintiles were also positively associated with the number of TCAM therapies used during therapy (*P* < .001). Parents who reported strong beliefs in TCAM were expected to have a 3.66 times higher rate of TCAM use than parents with beliefs in the bottom quintiles (incident rate ratio, 3.66; 95% CI, 1.97 to 6.80; *P* < .001; Fig 3B). A similar observation was made among adolescents’ beliefs in TCAM. For every unit of increase on the patient belief scale, the number of TCAM therapies used increased by a factor of 3.91 (incident rate ratio, 3.91; 95% CI, 2.28 to 6.73; *P* < .001).

## DISCUSSION

To our knowledge, this study is the first to report the prevalence, determinants, and beliefs of TCAM use among children with cancer in South America. Despite their proximity, we found significant differences in the frequency, types, and determinants associated with the TCAM use between Argentina and Uruguay. The results indicate that use of TCAM in Argentina is similar to prevalence data reported from high-income countries, with approximately 47% of participants reporting TCAM use, whereas in Uruguay, TCAM use is higher than that reported in low- and middle-income countries at 76% versus 66%, respectively.^3^

Consistent with the literature,^2,3^ we found that a high proportion of children use TCAM alongside cancer therapy. Despite the several similarities between both countries, the prevalence of TCAM use was significantly lower in Argentina than in Uruguay. This finding is of particular interest for Uruguay where its economic status is beginning to reflect that of high-income countries, yet TCAM use reflects that of low- and middle-income countries. The disparity in the use of TCAM between countries is likely due to institutional, ethnic, or cultural differences. Both countries have evolved economically but still encounter limited resources and lack trained personnel.^17,18^ Of note, cost was higher in Argentina than in Uruguay, which may reflect the preferred types of TCAM therapies or access to traditional remedies. A thorough understanding of the factors that contribute to variations in TCAM use will be important as policy, research, clinical, and educational guidelines evolve.

The majority of TCAM users disclosed their use of TCAM to their oncologist, a finding that has not been consistently reported in the literature. Clinicians in some areas of South America often are reluctant to ask about unconventional medicine and have struggled to adapt to these shifts in culture and patient demands. Moreover, medical schools in South America do not offer courses in TCAM therapies; consequently, most physicians are not prepared to discuss issues related to TCAM. Increased attention to the widespread use of TCAM and educational efforts aimed at all clinical disciplines may promote collaborations and information exchange among medical staff, TCAM providers, and families.

Most respondents reported that the use of TCAM primarily is for supportive care. In Argentina, use is largely linked to faith and cure, whereas in Uruguay, it is primarily for supportive care. This finding may be due to differences in religious affiliations between the countries or variations of the claims of traditional medicines within the context of cancer care. In either case, the finding underscores that a large proportion of families use TCAM alongside conventional therapy, and increased awareness is necessary to ensure safe integration of the two medical modalities. Several models for the integration of TCAM within oncology have been established, including within institutions in Argentina^19^; however, the optimal model for pediatric cancer units, particularly for units located in a low- and middle-income setting, still remains to be determined.

Also to our knowledge, this study is the first to report demographic variables and parental/patient beliefs associated with TCAM use in South America. The finding that the wealth index predicts the number of TCAM therapies is important for targeting families with a high likelihood of TCAM use. We also report for the first time that the strength of parental beliefs in TCAM therapies is a strong predictor of use and the number of TCAM therapies. We believe that this finding is significant because it suggests that educational initiatives be designed to target family beliefs about TCAM rather than lead to a cursory discussion about the risks, potential benefits, and costs of the therapies themselves. One approach may be to address families’ beliefs about psychosocial or nursing care through structured counseling and educational initiatives.

The study has several limitations. The surveys were provided to children and their families in a medical setting; thus, we do not know whether the use of TCAM may hinder access to conventional therapy or increase abandonment of therapy. We also do not know whether TCAM use is similar across the countries and regions because the size and diversity of Argentina is significantly different from that of Uruguay, and access to health services is likely to vary from one region to another. Finally, the cross-sectional nature of the study limits our understanding of how TCAM use fluctuates during various phases of therapy. Because of the limited sample size, we were unable to determine whether use of TCAM is higher among children with less-favorable prognosis or among those in palliative care.

In summary, more education and open communication are needed to improve the quality of health care and provide better transparency and mutual understanding of the use of TCAM among children with cancer. As health care providers in countries with limited resources, our role is to apply the best care based on the best available evidence. This study underscores the need to further investigate the effectiveness of TCAM in children and demonstrate cost-effectiveness so that patients, families, and physicians can make informed choices. More research is needed to integrate conventional medicine and TCAM use. As an initial step, this study identified predictors of TCAM use to target through educational initiatives.

## Figures and Tables

**Table A1:**
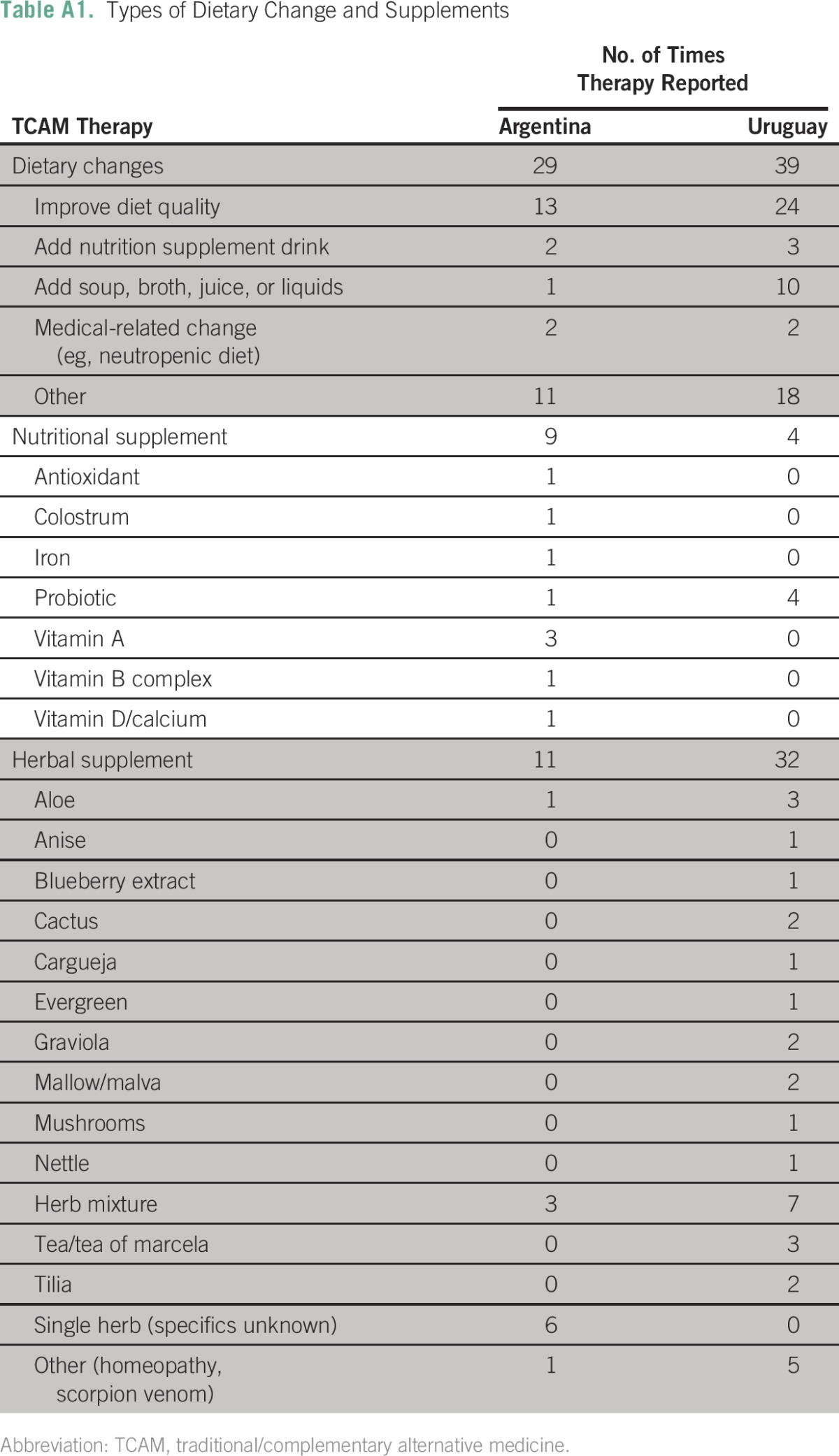
Types of Dietary Change and Supplements
